# Development and Validation of an Information Leaflet on Oral Care for Irradiated Patients

**DOI:** 10.2147/PPA.S256990

**Published:** 2020-10-06

**Authors:** Helene Bacher, Ramona Schweyen, Dirk Vordermark, Bernd Leplow, Jeremias Hey

**Affiliations:** 1 Department of Dental Prosthetics, University Hospital Halle/Saale, Halle (Saale), Germany; 2 Clinic for Radiotherapy, University Hospital Halle/Saale, Halle (Saale), Germany; 3 Institute of Psychology,Martin Luther University Halle-Wittenberg, Halle (Saale), Germany

**Keywords:** patient education, cancer, surveys and questionnaires, oral hygiene, information dissemination, patient satisfaction

## Abstract

**Purpose:**

The aim of the study was to develop an optimally designed and comprehensibly formulated patient information leaflet (PIL) to improve patients’ memory of information provided by physicians during a radiotherapy (head and neck area) consultation. This PIL was tested on unaffected probands for its usefulness in clinical practice.

**Patients and Methods:**

A panel of experts compiled the main topics using Lawshe’s content validity ratio. Flesch’s Reading Ease Score (FRE) and the Baker Able Leaflet Design (BALD) index were adapted to appropriate values to determine text comprehensibility and graphic design. The evaluation involving unaffected participants (231 men, 380 women, 21 not specified; mean age = 32 ± 13.63 years, range = 18–79 years) was conducted based on three questionnaires for four groups of respondents with varying prior knowledge of the subject. When answering the questionnaires, only half the participants had access to the PIL.

**Results:**

The expert panel included 59 out of 75 proposed topics. After reformulations, the FRE was adjusted from 38.5 to 51.4. The BALD index ranged from 24 to 26, depending on the printout edition. The evaluation of 632 unaffected participants indicated a difference in the correctly answered items that ranged from 2.86% to 30.76% between participants with and without access to the PIL.

**Conclusion:**

The general guidelines for the design of written patient information material were met. The evaluation of unaffected volunteers resulted in an advantage by answering the questionnaires after receiving the PIL. This study supports health practitioners in the development, design, and evaluation of written information material using scientific methods. An evaluation should be performed on affected patients.

## Introduction

Providing comprehensive medical information is essential to actively involve patients in the decision-making process on health issues. Doctor-patient consultations are the basis for all therapeutic interventions.^1^ The verbal communication of information to patients is of major importance because it is directly tailored to their current needs and gives them the opportunity to ask concrete questions.^2^–^4^ Disadvantages, such as misunderstood information and resulting uncertainties, can arise from a lack of communication between doctors and patients.^5^ Additionally, there are misconceptions about the ability of patients to receive information from consultation with physicians, to understand technical terms, and to follow the information flow.^6^,^7^ Studies show that the use of patient information leaflets (PILs) as a support in doctor-patient consultations leads to increased knowledge compared to doctor-patient consultations without leaflets.^8^,^9^

Providing patients with written information material has advantages for physicians and patients. PILs can help prevent misunderstandings and ensure the communication of information independently of the doctor. The use of PILs in combination with doctor-patient consultation improves patients’ memory of essential information, satisfaction, and compliance.^6^ Additionally, written information can be used for an unlimited number of times as a reference to remember relevant topics during the treatment process.^2^

Therefore, the optimal design of PILs is necessary to communicate pertinent information effectively.^10^,^11^ Because of these positive characteristics, PILs are created for many medical branches; however, they are often published without prior evaluation and vary significantly in quality.^12^ Ideally, PILs should be designed and pre-examined according to scientific guidelines.^11^ Consequently, in cooperation with several university hospitals, we developed a PIL concerning oral and dental care in the context of radiotherapy for the head and neck area and tested its usability and information provision. Its design and readability were examined based on scientific guidelines and the knowledge gain was evaluated on groups of probands with varying prior knowledge about the topic.

## Patients and Methods

### Design of the PIL

#### Content

A panel of experts—12 physicians (oral and maxillofacial surgery and radiotherapy) and dentists from different university hospitals—created the PIL content. To compile relevant topics regarding PIL content, recent literature in German and English concerning prophylaxis and side effects of radiotherapy of the head and neck area was analyzed, thematically subdivided, and summarized. The relevance of the main topics was determined in a referendum of the panel of experts using Lawshe’s method.^13^

##### Flesch’s Reading Ease Score (FRE)

After the contents of the PIL had been formulated, their readability was determined using the FRE for texts in German.

#### PIL Design

The leaflet was designed in accordance with guidelines for the design of PILs with Adobe Illustrator (Adobe Systems Software Ireland Limited, Dublin, Ireland) to enable the print of the PIL in different formats. Subsequently, the PIL was evaluated by a third person using the BALD index;^14^ for this purpose, certain design elements concerning layout, font, line spacing, images and choice of words were scored. The maximum number of points was 32.

### PIL Evaluation

#### Questionnaires

Three different questionnaires (A–C) were developed to determine the amount of information conveyed by the PIL. The main topics of questionnaire A (21 items) were dental pre-treatment, side effects during radiotherapy, and handling of protheses during radiotherapy. Questionnaire B (19 items) covered handling the radiation splints, mucositis, and dental care concerning radiotherapy. Questionnaire C (18 items) included xerostomia, nutrition during radiotherapy, and dental care concerning radiotherapy.

The items were available as hypotheses, which could be answered by the probands with “right” or “wrong.” To determine the questionnaire characteristics, the index of difficulty, the variance, and the item-total correlation were calculated. Pearson-Bravais was used for correlation coefficients. Cronbach’s α was used to define the internal consistency of the questionnaires. Questionnaire evaluation was conducted on the groups that had no access to the PIL.

#### Probands

Groups of probands with different levels of prior knowledge about the topic and the contents of the PIL were interviewed. The minimum number of participants per group were 20, based on the previous sample size calculation. Exclusion criteria for all groups were completed medical studies and/or a planned, current or completed radiotherapy in the head and neck area. Group 1 included dentistry students in clinical (6th–10th) semesters. Group 2 included students of dentistry in pre-clinical (1st–5th) semesters and excluded students of group 1. Group 3 consisted of students from other disciplines who had not completed or begun medical or dental studies. Group 4 included patients of the Clinic for Dental Prosthetics of the University Clinic Halle-Wittenberg who also did not meet the inclusion criteria of the other groups.

All students interviewed were enrolled in a course of study at Martin Luther University, Halle-Wittenberg, Germany. Participants were recruited by a person not involved in the study process (groups 1–3) or in the treatment process (group 4). Prior knowledge of the topic was rated highest in group 1 and lowest in group 4.

The investigations were conducted between February 2012 and March 2013 in cooperation with the Institute of Psychology of the Martin Luther University Halle-Wittenberg. Half the participants in each study group were given a PIL by random allocation. Thereafter, 24–96 h later, a randomly assigned questionnaire was distributed to all probands. No personal data were recorded. Consent to voluntary participation resulted in submitting the anonymously completed questionnaire at a time independent of the receipt of the questionnaire. Owing to the complete anonymity, it was impossible to withdraw from the study after questionnaire submission.

The study was conducted in accordance with the Declaration of Helsinki (2013) and based on the conduct of medical research in accordance with ethical principles. The medical faculty’s ethics committee at the Martin Luther University Halle-Wittenberg approved the study protocol for the subsequent investigation on actually affected patients (reference number 2017–119). Because it was a one-time, completely anonymous pre-trial of uninvolved probands no ethics vote was required.

#### Data Analysis

The data from the evaluation of the PIL were digitalized and analyzed using SPSS (IBM, Ehningen, Germany) and Excel (Microsoft, Redmond, USA). To check the data for normality, the Kolmogorov–Smirnov test with significance correction according to Lilliefors was performed. Furthermore, *t*-tests, Mann–Whitney *U*-tests, and Kruskal–Wallis tests were performed to calculate significant differences. A p-value below 0.05 was considered significant.

## Results

### PIL Design

#### Content

The panel of experts voted on the previously proposed topics. Of the 75 topics, 59 were included in the PIL content. The main topics compiled by the panel of experts and their evaluation based on Lawshe’s procedure were the indication for radiotherapy, the side effects of the radiotherapy and how to avoid them and how to deal with them, the procedure of the dental pre-treatment, and the care of the mouth during and after radiotherapy. Excluded were topics such as the medical basics of radiotherapy and organizational details (Table 1). When topics were rated very relevant by panelists, they were formulated in more detail in the PIL and highlighted with the help of high-contrast text fields and associated graphic illustrations.

**Table 1:** Results of the Panel of Experts’ Voting on the Main Topics of the Patient Information Leaflet

Topic	Essential	Useful, but Not Essential	Irrelevant	Content Validity Ratio
Medical basic knowledge of radiotherapy	7	2	3	0.16
Indication for radiotherapy	11	1	0	0.83
Combination of radiotherapy with operation or chemotherapy	6	2	4	0
Side effects	12	0	0	1
Xerostomia	12	0	0	1
Taste loss	12	0	0	1
Mucositis	12	0	0	1
Increased risk for candida infections	9	2	1	0.5
Radiation caries	12	0	0	1
Osteoradionecrosis	12	0	0	1
Dental pre-treatment				
Extraoral examination	9	1	2	0.5
Dental examination	12	0	0	1
Dental radiograph	12	0	0	1
Professional dental cleaning	12	0	0	1
Impressions for radiation splints	12	0	0	1
Dental pre-therapy (fillings, deep scaling, etc.)	8	3	1	0.33
Indications for teeth extractions	12	0	0	1
Importance of daily oral hygiene	12	0	0	1
Dental care during radiotherapy				1
Regular even in cases of sulcus bleeding and pain	12	0	0	1
Gum line	12	0	0	1
Interdental spaces	12	0	0	1
Soft toothbrush	12	0	0	1
Palate/tongue massage	12	0	0	1
Possibility to clean teeth without toothpaste	11	1	0	0.83
Cleaning methods	8	2	2	0.33
Cleaning the toothbrush with Chlorhexidine solution	12	0	0	1
Handling removable dentures				
No dentures during the radiotherapy	12	0	0	1
Handling/storage	12	0	0	1
Start of dental rehabilitation three months after completion of therapy	11	0	0	0.83
Radiation splints as radiation protection				
Radiation scatter	12	0	0	1
Cleaning/care/storage	11	1	0	0.83
Handling	11	1	0	0.83
Radiation splints for fluoride application				
Effect	12	0	0	1
Fluoride application	12	0	0	1
Period of application	11	1	0	0.83
Handling	10	1	1	0.67
Cleaning/care/storage	10	2	0	0.67
Restoration by the dentist if necessary	8	4	0	0.33
Mucositis				
Adequate dental care	12	0	0	1
Mouth rinses with water, saline solution, or sage tea	12	0	0	1
No chamomile	11	0	1	0.83
Ice cubes	12	0	0	1
Pineapple	12	0	0	1
Pilocarbin	8	1	3	0.33
Painkillers	8	4	0	0.33
Abstinence from alcohol and nicotine	9	3	0	0.5
Xerostomia				
Stimulate salivation	12	0	0	1
Water uptake	11	1	0	0.83
Saline flush	11	1	0	0.83
Sugar free candy and chewing gum	12	0	0	1
Saliva substitute	12	0	0	1
Vaseline for lips and corners of the mouth	11	1	0	0.83
Trismus				
Exercise	9	1	2	0.5
Physiotherapy	9	3	0	0.5
Nutrition				
Small meals	12	0	0	1
Water uptake	12	0	0	1
Vitamin A rich juices	12	0	0	1
Edible oil with fruit juices	12	0	0	1
Low acid fruit and vegetables	12	0	0	1
Milk products (calcium)	12	0	0	1
More fish than meat	12	0	0	1
Fibers	11	0	1	0.83
Mild spices	10	0	2	0.67
Tube feeding if necessary	9	2	1	0.5
Aftercare				
Appointments with radiotherapists/operation aftercare/dental follow-up	8	2	2	0.33

**Note:** Dark shaded fields indicate topics that have not been included in the content of the PIL.

#### Readability Score

After analysis of the information from the literature, the readability score for German language was 38.5. By reformulating and adapting the choice of words, the readability score was changed to 51.4 (Table 2).

**Table 2:** Readability Score (FRE: Flesch-Reading-Ease)

	Sentences	Words	Syllables	*FRE_German_*
Version 1	94	1.313	2.863	38.5
Version 2	127	1.254	2.548	51.4

#### Design

##### Design Characteristics

The design of the PIL was based on current guidelines for the design of patient information material. Vector graphics were used to enable printing in various formats. The layout was prepared in several colors varying between main text, additional information and very relevant sections. There were 20 multi-colored schematic illustrations and four black and white pictograms. These were placed next to the corresponding text passages.

##### BALD Index

The BALD index of the PIL depends on its print format. The BALD index is 24 in size A6, and 26 in size A4 (Table 3).

**Table 3:** Design Characteristics of the Patient Information Leaflet

Design Characteristics	Value	
	A6	A4
Lines 50–89 mm	1	0
Separation between lines	1	3
Lines unjustified	1	1
Serif typeface	0	0
Type size	2	3
First line indented	0	0
Titles (headings) lower case	1	1
Italics	0	0
Positive advice (“do” instead of “do not”)	2	2
Headings stand out	2	2
Numbers are all Arabic	1	1
Boxed text	1	1
Pictures (not including cover pictures)	3	3
Number of colors	3	3
White space	3	3
Paper quality	3	3
Total	24	26

### Evaluation of the PIL

#### Questionnaires

The average item difficulty was 0.63. The average variance of the items was 0.184. The average index of discrimination was 0.18. Cronbach’s αs for questionnaires A–C were 0.786, 0.739, and 0.611, respectively.

#### Evaluation

##### Descriptive Statistics

The number of probands was split up among groups of different levels of prior knowledge (Table 4). Table 5 presents an overview of the descriptive statistics of the study population. The mean age differed significantly between all groups (Analysis of variance; p< 0.001, Levene’s test; p< 0.001; post hoc Bonferroni p< 0.05).

**Table 4:** Groups and Number of Probands

Group	Questionnaire A		Questionnaire B		Questionnaire C		Total
	Access to PIL	No Access to PIL	Access to PIL	No Access to PIL	Access to PIL	No Access to PIL	
1	25	25	25	25	26	24	**150**
2	26	25	27	27	23	26	**154**
3	23	22	30	27	30	26	**158**
4	29	24	30	26	30	31	**170**
**Total**	**103**	**96**	**112**	**105**	**109**	**107**	**632**

**Abbreviation:** PIL, patient information leaflet.

**Table 5:** Descriptive Statistics of the Probands

Group	Age		Sex					
			Male		Female		No information provided	
	M	SD	n	%	n	%	n	%
Dentistry students	24.66	3.53	122	40.1	176	57.9	6	2.0
Students from other disciplines	29.10	11.67	29	18.4	119	75.3	10	6.3
Patients	46.73	15.70	80	47.1	85	50.0	5	2.9
Total	31.96	13.63	231	36.6	380	60.1	21	3.3

**Abbreviations:** PIL, patient information leaflet; SD, standard deviation; n, number.

##### Testing of Knowledge

**Figure 1:**
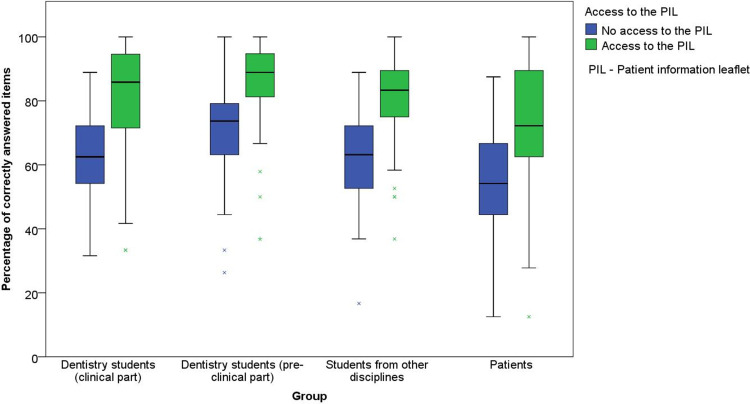
Percentage of correctly answered items on questionnaires A, B, and C.

The mean numbers of correctly answered items for questionnaires A–C are shown in Table 6. Figure 1 shows the results of the three questionnaires relating to respondents’ previous knowledge. The average difference between the groups with and without access to the PIL was 18.4%. In all groups of probands, a high percentage of correctly answered items was evident when access to the PIL was provided. In questionnaire C, the difference to the group without access to the PIL was non-significant in the groups of patients and dental students in pre-clinical semesters. The previous knowledge of the different groups had an influence on the percentage of correctly answered items (Kruskal–Wallis test; p < 0.001), both in the group with access to the PIL (Mann–Whitney *U*-test; p < 0.001) and in the group without access to the PIL (Mann–Whitney *U*-test; p < 0.001).

**Table 6:** Percentage of Correctly Answered Items

Group	No Access to the PIL		Access to the PIL		Mean Difference	p
	Percentage of Correctly Answered Items	SD	Percentage of Correctly Answered Items	SD		
Questionnaire A						
1	76.17	9.04	83.50	10.58	7.33	< 0.001
2	59.38	15.46	84.03	16.61	24.65	< 0.001
3	62.12	17.15	77.54	13.57	15.45	0.002
4	52.43	16.62	83.19	19.37	30.76	< 0.001
Questionnaire B						
1	64.42	12.65	87.79	15.69	23.37	< 0.001
2	57.69	11.19	88.11	12.77	30.42	< 0.001
3	54.58	9.92	81.05	13.30	26.47	< 0.001
4	48.99	13.87	75.44	13.28	26.45	< 0.001
Questionnaire C						
1	73.61	15.65	88.89	9.56	15.28	< 0.001
2	68.80	12.77	71.74	18.12	2.94	0.436
3	69.26	10.18	83.98	9.34	14.72	< 0.001
4	58.07	15.64	60.93	13.80	2.86	0.258

**Abbreviations:** PIL, patient information leaflet; SD, standard deviation; p, p-value.

## Discussion

### PIL Design

#### Content

Clinical studies show that patients can take in only a limited amount of information from consultations with doctors.^15^ Factors such as high psychological stress and increased stress levels have a negative effect on recall.^16^ Contrastingly, providing written information in addition to verbal information can positively influence information communication.^8^,^9^

However, this requires the provision of an appropriate amount of information. The scope of PILs varies and can range from a single page to multi-part booklets.^12^ The investigated PIL was arranged on 12 pages. This allows for a manageable reading time of about 10 minutes. As prepared, the information can ideally serve both as therapy preparation and as support in dealing with short- and long-term side effects. If the doctor providing the information uses the PIL during the consultation, it serves both as illustrative material and as a guide to address all relevant topics. Furthermore, the information material can be provided to patients prior to consultations, thus allowing time to prepare questions and stay engaged in the discussion.

However, during the discussion of relevant topics by panelists from different fields, the amount of information was reduced to only the essential aspects. When reducing the written information, it must be remembered that it is always combined with a medical consultation. Thus, there is no claim to completeness; however, the effort to convey essential information in an appropriate scope is maintained.

#### Readability Score

A readability index quantifies how easily text is read. In general, an increase in readability leads to a better memory of the content of the text and a shorter reading time.^7^ Difficult text generally has an index of 30–50, while medium difficulty text is between 50 and 70. In general, a higher readability index of over 70 is targeted for comparable PILs.^17^

The readability index of the original version of the text was originally 38.5. We obtained an index of 51.4 by rewording and changing the choice of words. When using this index to optimize text, the comprehensibility and readability of text are not always directly correlated. Text comprehension, which is more important than readability per se, is influenced by other factors such as readers’ competence, motivation, interest in the subject matter, and prior knowledge.^7^ The reading competence of the intended target group should not be underestimated; otherwise, readers with a high level of reading competence might be disinterested in the information. Motivation to the PIL should be rated as high, as the information has been adapted the target group. Adequate prior knowledge of the readers can also be expected through the combination with the medical clarification interview. In this context, the adapted readability index to the intended field of application of the information material was appropriate. Accordingly, the readability index should always be combined with the individual assessment by an expert reader and should not be used as the sole indicator of text comprehensibility.^18^

#### Design

After the elaboration and formulation of relevant topics for the PIL, an appropriate form is essential to arouse the interest of the target group. The graphic representation of important facts was used to make them easier to comprehend by abstraction. In other studies, pictograms led to improved information recall^19^. The design of the PIL was adapted to the capacity of different readers to absorb information according to topic relevance. The BALD index was applied to the two proposed formats, which displayed above-average results.^20^

### Evaluation of the Questionnaires

#### Questionnaires

The questionnaire characteristics followed the recommended guidelines for statistical testing.^21^ The internal consistency of the questionnaires is relatively low. Considering the relatively ease simplicity of some of the items of the questionnaires that could be answered correctly by many participants, the low Cronbach’s alpha can be explained. These items were intentionally left in the questionnaire because the main purpose was to convey basic knowledge rather than to distinguish between educational marks, which would be the aim of classical examinations.

#### Evaluation

The mean age of randomly selected non-affected patients was lower than the mean age of patients previously treated with head and neck radiation (women = 66.2 yr, men = 63.8 yr).^22^ Similarly, most patients surveyed were women, contrasting the large proportion of male patients with head and neck cancer.^22^ Although our sample differs from cases, a certain comparability of the ability to remember information from written material is assumed. Although the interviews were conducted with German-speaking patients, a general transfer of the procedure compared to the procedure in other regions is possible within our limitations.

The evaluation of the participants showed a higher percentage of correctly answered items in all groups with access to the PIL, confirming the benefit of written information material.^8^,^9^,^12^ The mean difference of the group of the unaffected patient group was comparable to that of similarly structured studies.^23^ The difference in the average percentage of correctly answered items between the groups with and without access to the PIL varied between the questionnaires. In the first two questionnaires, a clear difference was noticeable; for questionnaire C, no significant difference was found between groups 2 and 4. Possibly the topic “nutrition” in questionnaire C could have influenced the result. While the other topics were specifically related to the course of radiotherapy, this topic is also part of general education. Participants’ attitude toward this topic is conditioned by psychosocial influences; therefore, the items were not answered only with the help of the knowledge gained from the PIL.^24^ The prior knowledge of the groups had an influence on the percentage of correctly answered items, both in the group that had received the PIL and in the group that had not. Our findings indicate that the PIL is helpful in conveying knowledge and, in combination with the prior knowledge from doctor-patient consultations, it improves patients’ comprehension of information even further.

## Conclusion

Although PILs are created for many medical fields, they are often published without prior evaluation and vary considerably in quality. The developed PIL was created in accordance with the requirements of the guidelines for the design of patient information material. Among the groups of participants without previous specific knowledge, the average difference in the percentage of correctly answered items with and without access to the PIL was 19.4%. This PIL can therefore be used as an adequate supplement to educational consultations. This study shows the great importance of pre-evaluation for the implementation of an adequate PIL and informs health practitioners in the preparation, design and evaluation of written information material. An evaluation of patients treated with radiotherapy in the head and neck area should follow.
